# A Brief Review of Chimera State in Empirical Brain Networks

**DOI:** 10.3389/fphys.2020.00724

**Published:** 2020-06-30

**Authors:** Zhenhua Wang, Zonghua Liu

**Affiliations:** School of Physics and Electronic Science, East China Normal University, Shanghai, China

**Keywords:** synchronization, chimera state, order parameter, neuronal system, cerebral cortex, 89.75.Hc, 05.45.Xt, 68.18.Jk

## Abstract

Understanding the human brain and its functions has always been an interesting and challenging problem. Recently, a significant progress on this problem has been achieved on the aspect of chimera state where a coexistence of synchronized and unsynchronized states can be sustained in identical oscillators. This counterintuitive phenomenon is closely related to the unihemispheric sleep in some marine mammals and birds and has recently gotten a hot attention in neural systems, except the previous studies in non-neural systems such as phase oscillators. This review will briefly summarize the main results of chimera state in neuronal systems and pay special attention to the network of cerebral cortex, aiming to accelerate the study of chimera state in brain networks. Some outlooks are also discussed.

## 1. Introduction

Network physiology is a new field initiated by Ivanov et al. in 2012, aiming to reveal the relationship between network topology and physiologic function (Bashan et al., 2012; Ivanov and Bartsch, 2014; Bartsch et al., 2015; Ivanov et al., 2016). Generally, a physiological network is consist of different physiologic organ systems, such as the dynamical network of six physiologic systems with nodes being brain activity (EEG waves: δ, θ, α, σ, β), cardiac (HR), respiratory (Resp), chin muscle tone, leg, and eye movements. In contrast to a static complex network, the topology (i.e., number and strength of network links) of physiological network usually vary with time, resulting in different physiologic states such as different sleep stages [deep, light, rapid eye movement (REM) sleep, and quite wake]. That is, each physiological state of physiological network corresponds to a specific network structure. The transition from one physiologic state to another is associated with fast reorganization of physiological interactions. Network physiology can be successfully applied to explain phase synchronization between organ systems (Chen et al., 2006; Xu et al., 2006; Ivanov et al., 2009; Bartsch et al., 2012; Bartsch and Ivanov, 2014). A typical system of network physiology is the brain network, which is the focus of this review.

One of the most challenging and long standing problems is the understanding of the powerful brain functions such as data processing, function approximation, and pattern recognition etc., which has been considered as a black box for a long time. To solve this problem, it is necessary to figure out the brain network first. So far, it is well known that the human cerebral cortex is a huge network, consisting of 10^11^ neurons and 10^14^ links. Thus, it is almost impossible to figure out the structure of this huge network. Fortunately, owing to the modern physical detections such as electroencephalogram (EEG), magnetic resonance imaging (MRI), magnetoencephalogram (MEG), and diffusion tensor imaging (DTI) etc., we can now conveniently obtain a coarse-grained brain network with a finite size of hundreds to thousands nodes. It has been found that the obtained brain network is of a modular structure with a complex connectivity (Hilgetag and Kaiser, 2004), which provides a base for the inherent parallel nature of brain computations (Meunier et al., 2010; Zamora-Lopez et al., 2010). The quantification of complexity in brain networks can be also measured by the multifractality and other factors (Liu et al., 2015; Lin et al., 2016; Xue and Bogdan, 2017; Racz et al., 2018; Gupta et al., 2019; Yang et al., 2019). A larger or more precise brain network can be obtained by reconstructing the connectivity under partial observability assumptions, which is common to many real world settings or experiments (Gupta et al., 2018; Xue and Bogdan, 2019).

By the physical detections, it is revealed that synchronization of neuronal ensembles in the network of cerebral cortex is the base of various neurobiological processes. For example, the alpha synchronization is task-related and is also associated to top-down processing (Benedek et al., 2011). Further, it is pointed out that synchronization and desynchronization of neural activity are closely related to both the normal functions of brain and its disorders, such as epileptic seizures and Parkinson's disease etc. (Rothkegel and Lehnertz, 2014). For instance, during an epileptic seizure, some regions of brain are strongly synchronized but the others are desynchronized (Ayala et al., 1973). While in Parkinson's disease, synchronized activity is absent in the brain regimes of damaged neurons (Levy et al., 2000).

Moreover, it was found that the synchronized and desynchronized behaviors are usually co-existed in the time series of brains. For example, the EEG data showed that during sleep of dolphin, its two brain hemispheres have independently synchronized and desynchronized behaviors at the same time (Mukhametov et al., 1977), i.e., one hemisphere is in sleep and another remains awake. This phenomenon is called as *unihemispheric slow-wave sleep* and has been also found in other aquatic animals and migrated birds (Rattenborg et al., 2000). On the other hand, the similar phenomenon was reported in the context of neuroscience, called *bump state*, where the firing rate is higher at some spatial locations but a constant at other spatial positions (Laing and Chow, 2001). This bump state takes an important role for feature selectivity in models of the visual system (Somers et al., 1995), the head direction system (Zhang, 1996), and working memory (Camperi and Wang, 1998).

To understand the mechanisms of these coexisted behaviors in brains, numerous efforts have been paid to the synchronization of coupled oscillators. One of its recent progresses is chimera state, which is closely related to the phenomenon of unihemispheric sleep. Chimera state represents the coexistence of coherent and incoherent dynamics. It is surprising that this behavior occurs in symmetrically coupled identical oscillators. This counterintuitive phenomenon was first discovered in 2002 (Kuramoto and Battogtokh, 2002) and then named in 2004 (Abrams and Strogatz, 2004). Since then, chimera state has become a hot topic and different kinds of chimera states have been revealed in different systems such as the chaotic dynamical systems (Omelchenko et al., 2012), time-delayed system Sethia et al. (2008), and systems with regular topology (Ko and Ermentrout, 2008; Yao et al., 2013; Tian et al., 2017) etc. Initially, chimera states were shown to emerge in systems of nonlocal coupling. Recently, it has been extended to the system of globally coupled oscillators (Chandrasekar et al., 2014), and even in complex networks (Zhu et al., 2014). Further, to explain the alternating activity patterns between the hemispheres over time (Mukhametov et al., 1977), Ma et al. considered the effect of time-delay in two coupled populations and found that the synchronous and desynchronous behavior do alternate between the two groups over time (Ma et al., 2010). At the same time, chimera states have also been implemented in several experiments such as on chemical oscillators (Tinsley et al., 2012), mechanical oscillators (Martens et al., 2013), electronic oscillators (Larger et al., 2013), electrochemical oscillators (Wickramasinghe and Kiss, 2013; Schmidt et al., 2014), and optoelectronic oscillators (Larger et al., 2015). See reviews Panaggio and Abrams (2015) and Majhi et al. (2019) for details.

In sum, chimera states are mainly studied on phase oscillators. As chimera state may represent the mechanism of unihemispheric sleep where the neurons in the sleepy hemisphere are synchronized and the neurons in the awake hemisphere are desynchronized, it is necessary to pay more attention on the chimera state in neural systems. Fortunately, some interesting results have already been obtained in this line, which involve most typical neural models. For examples, chimera states have been studied in leaky integrate-and-fire neurons (Olmi et al., 2010), Morris-Lecar neurons (Calim et al., 2018), FitzHugh-Nagumo neurons (Omelchenko et al., 2013, 2015), Hindmarsh-Rose neurons (Hizanidis et al., 2014, 2016), and Hodgkin-Huxley neurons (Sakaguchi, 2006; Glaze et al., 2016). To accelerate the study of chimera state in brain networks, it is necessary to systematically summarize the measures of chimera state and its recent progress in empirical brain systems, which has not been paid enough attention in the previous reviews (Panaggio and Abrams, 2015; Majhi et al., 2019). Thus, we here briefly summarize the main results of chimera state in neuronal systems and pay a special attention to the network of cerebral cortex.

## 2. Three Measures of Chimera State

To characterize the chimera state, three statistical measures have been proposed so far, by using the time series of network. The first measure is the order parameter by

(1) $$\begin{array}{l} \rho \left(t\right)e^{i\Phi \left(t\right)}=\frac{1}{N}\overset{N}{\underset{j=1}{\sum }}e^{i\theta_{j}\left(t\right)}, \end{array}$$

where ρ represents the phase coherence of oscillators, Φ(*t*) is the average phase of all oscillators, θ_*j*_(*t*) is the phase variable of the *j*-th oscillator. The system is a complete synchronization when ρ = 1 and a complete desynchronization when ρ = 0. This measure can be conveniently used to the system consisting of two groups. It is a chimera state when one group has an order parameter ρ ≈ 1 and the other ρ ≈ 0.

A key element of this measure is to calculate the phase variable θ_*i*_(*t*). However, there is not such an explicit variable in all the neuronal models, in contrast to the Kuramoto phase oscillator. Generally, there are two ways to solve this problem. The first one can be used to a general nonlinear oscillator not necessarily having a well-defined rotational center. Let *u*_*i*_ and *v*_*i*_ represent two variables of the *i*-th neuron, respectively. The phase θ_*i*_(*t*) can be calculated as (Osipov et al., 2003; Liu et al., 2009)

(2) $$\begin{array}{l} \theta_{i}\left(t\right)=\arctan\left(\frac{{\dot{v}}_{i}}{{\dot{u}}_{i}}\right), \end{array}$$

where ${\dot{v}}_{i}$ and ${\dot{u}}_{i}$ denote the derivatives of *u*_*i*_ and *v*_*i*_, respectively. The second one is to calculate the phase θ_*i*_(*t*) by

(3) $$\begin{array}{l} \theta_{i}\left(t\right)=\arctan\left(\frac{v_{i}}{u_{i}}\right), \end{array}$$

provided that *u*_*i*_ and *v*_*i*_ move around the origin. An alternative way of Equation (3) is to calculate its instantaneous angular frequency (Pereira et al., 2007) by

(4) $$\begin{array}{l} {\dot{\theta }}_{i}=\frac{{\dot{v}}_{i}u_{i}-{\dot{u}}_{i}v_{i}}{u_{i}^{2}+v_{i}^{2}}. \end{array}$$

Then, the phase θ_*i*_ can be integrated from Equation (4).

The second measure is based on the first one but only for the networks having more than two communities (Shanahan, 2010). Consider a network with *m* communities. We let ρ_*i*_(*t*)(*i* = 1, …, *m*) represent the order parameter for each community *i* at time *t*. A chimera state means that the values of ρ_*i*_(*t*) for different communities are not the same. Based on this feature, two indices are introduced to measure the chimera state, i.e., the index of the metastability λ and the chimera-like index χ. For the former, we first calculate ρ_*i*_(*t*) for *T* points, i.e., *t* ∈ {1 … *T*}. Then, their variance can be obtained as

(5) $$\begin{array}{l} \sigma_{met}\left(i\right)=\frac{1}{T}\overset{T}{\underset{t=1}{\sum }}{\left(\rho_{i}\left(t\right)-{\langle \rho_{i}\left(t\right)\rangle }_{T}\right)}^{2}. \end{array}$$

σ_*met*_(*i*) gives the fluctuation of synchronization in the community-*i*, i.e., the metastability. The index of the metastability for the entire network is

(6) $$\begin{array}{l} \lambda ={\langle \sigma_{met}\left(i\right)\rangle }_{m}. \end{array}$$

Similarly, an instantaneous variance over all the *m* communities can be introduced as (Shanahan, 2010)

(7) $$\begin{array}{l} \sigma_{chi}\left(t\right)=\frac{1}{m}\overset{m}{\underset{i=1}{\sum }}{\left(\rho_{i}\left(t\right)-{\langle \rho_{i}\left(t\right)\rangle }_{m}\right)}^{2}. \end{array}$$

Then, the chimera-like index χ is

(8) $$\begin{array}{l} \chi ={\langle \sigma_{chi}\rangle }_{T}. \end{array}$$

Thus, the chimera-like χ and metastability λ indices quantify the degree of synchronization along time and among communities, respectively.

**Figure 1:**
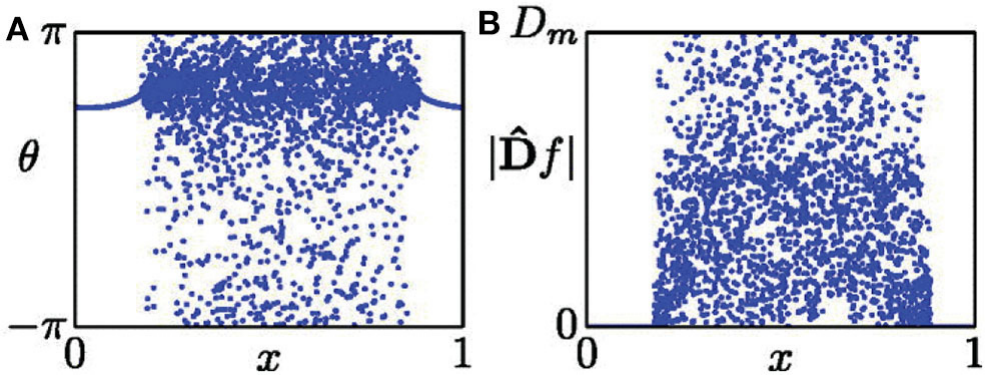
**(A)** Snapshot of a chimera state. **(B)** Distribution of |*Df*| from the data set of **(A)**. Reprinted with permission from Kemeth et al. (2016).

The third measure is for a general network with or without clear communities (Kemeth et al., 2016). Its idea is based on the local curvature for the spatial coherence, represented by the second derivative in the case of one spatial dimension. In this case of one dimension, the local curvature *D* can be calculated as

(9) $$\begin{array}{l} Df\equiv f\left(x+\Delta x,t\right)-2f\left(x,t\right)+f\left(x-\Delta x,t\right), \end{array}$$

where *f* represents the spatial data on a snapshot at time *t*. Figure 1 shows its schematic figure where (Figure 1A) is a typical snapshot of chimera state and (Figure 1B) is the mapped *D*, with *D*_*m*_ being the maximal value of |*Df*|. *D*_*m*_ represents the curvature of the oscillator with its two neighbors being shifted 180^*o*^ in phase (Kemeth et al., 2016). From Equation (9) we see that |*Df*| equals to zero in the synchronous regime and finite values with pronounced fluctuations in the desynchronized regime.

From Figure 1B, we see that |*Df*| is distributed between 0 and *D*_*m*_. Letting *g* be the normalized probability density of |*Df*|, *g*(|*Df*| = 0) is the fraction of spatially synchronous regimes. Thus, *g*(|*Df*| = 0) is unity for a complete synchronized state, zero for a complete desynchronized state, and a value between zero and unity for a chimera state. As numerical simulations have fluctuation, it was suggested that those points with |*Df*| < 0.01*D*_*m*_ should be considered as synchronized, and otherwise desynchronized (Kemeth et al., 2016). That is, the fraction of coherent regions can be calculated by

(10) $$\begin{array}{l} g_{0}\left(t\right)=\int_{0}^{\delta }g\left(t,|Df|\right)d|Df|, \end{array}$$

with δ = 0.01*D*_*m*_. Therefore, we have *g*_0_ = 1 for a complete coherent, *g*_0_ ≈ 0 for desynchronized, and 0 < *g*_0_ < 1 for chimera states.

Except these three main measures, Gopal et al. introduced another approach to characterize the chimera and multichimera states (Gopal et al., 2014). Their approach is based on a transformation from the framework of **x**_*i*_ to a new framework **z**_*i*_, *i* = 1, 2, ⋯ , *N*, where **z**_*i*_ = **x**_*i*_ − **x**_*i*+1_. In their approach, chimera states can be measured by two indices, i.e., the strength of incoherence and discontinuity measure. Although these measures are different each other, they are all robust in numerical simulations and can be applied in different systems.

## 3. Chimera State in Neuronal Systems

Although chimera state is mainly studied in phase oscillators, it has also been observed in other oscillators with amplitudes, including the neuronal systems. We here make a brief review on the results of chimera state in neuronal systems, mainly focusing on the FitzHugh-Nagumo (FHN) neurons and Hindmarsh-Rose (HR) neurons.

We first introduce the studies of chimera state in FHN neurons. In this aspect, Omelchenko et al. considered the case of FHN neurons by a stronger coupling and found a multi-chimera state (Omelchenko et al., 2013). For the sake of simplicity, they introduced a rotational coupling matrix and found that it is possible for the system to show both chimera and multichimera states. Their discussion is as follows. Consider *N* FHN neurons coupled nonlocally on a ring

(11) $$\begin{array}{l} \epsilon \frac{du_{k}}{dt}=u_{k}-\frac{u_{k}^{3}}{3}-v_{k}+\frac{\sigma }{2R}\overset{k+R}{\underset{j=k-R}{\sum }}[b_{uu}\left(u_{j}-u_{k}\right)\\ \;+b_{uv}\left(v_{j}-v_{k}\right)],\\ \frac{dv_{k}}{dt}=u_{k}+a_{k}+\frac{\sigma }{2R}\overset{k+R}{\underset{j=k-R}{\sum }}[b_{vu}\left(u_{j}-u_{k}\right)\\ \;+b_{vv}\left(v_{j}-v_{k}\right)],\;k=1,2,\cdots \;,N \end{array}$$

where *u*_*k*_ and *v*_*k*_ are the activator and inhibitor variables, respectively. ϵ is taken as ϵ = 0.05. A neuron is excitable for |*a*| > 1 and oscillatory otherwise (Omelchenko et al., 2013). For simplicity, Omelchenko et al. (2013) considered the case of identical neurons with *a*_*k*_ ≡ *a* ∈ (−1, 1). In the framework of Equation (11), the coupling strength σ is a constant within the *R* nearest neighbors from both sides but zero otherwise, marking the feature of nonlocal coupling. To observe chimera states in neuronal systems, Omelchenko et al. introduced the rotational coupling matrix (Omelchenko et al., 2013)

(12) $$\begin{array}{l} B=\left(\begin{array}{cc} b_{uu} & b_{uv}\\ b_{vu} & b_{vv} \end{array}\right)=\left(\begin{array}{cc} \cos\phi & \sin\phi \\ -\sin\phi & \cos\phi \end{array}\right), \end{array}$$

where ϕ is a parameter of coupling phase, representing the relative phase difference of interacting oscillators. This is a kind of cross-coupling. The value of chosen ϕ determines the property of coupling, i.e., attractive or repulsive.

**Figure 2:**
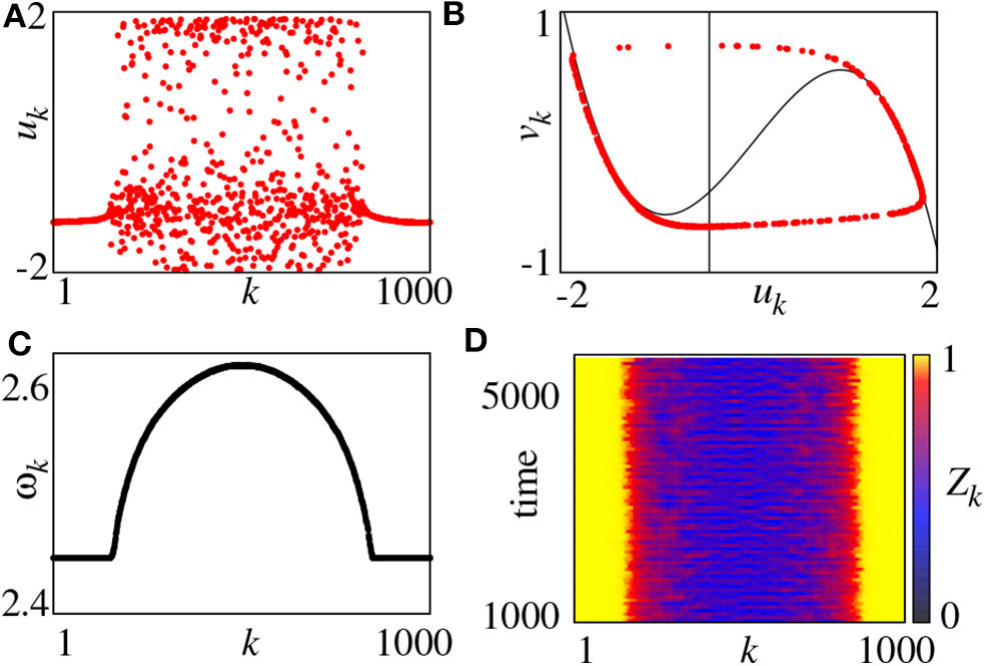
**(A)** Snapshot of the variables *u*_*k*_ for *t* = 5, 000, **(B)** snapshot in the (*u*_*k*_, *v*_*k*_) plane for *t* = 5, 000 (black lines denote the nullclines of the FHN system), **(C)** mean phase velocities ω_*k*_, **(D)** local order parameter *Z*_*k*_. Parameters: *N* = 1000, *r* = 0.35, σ = 0.1, *a* = 0.5, ϕ = π/2 − 0.1. Reprinted with permission from Omelchenko et al. (2013).

For convenience, the parameter *r* = *R*/*N* is used to represent the coupling radius. The system of Equation (11) has different behaviors for different *r* and σ. For example, for fixed *a* = 0.5 and ϕ = π/2−0.1, a chimera state can be observed for small coupling strength σ and multichimeras state for larger σ. Figure 2 shows the case of small σ where Figure 2A represents a snapshot of *u*_*k*_ at the moment of *t* = 5, 000. It is a typical chimera state consisting of coherent and incoherent parts. Figure 2B shows a further confirmation where the desynchronized part is distributed along the limit cycle.

On the other hand, we may also use the mean phase velocity of oscillators to characterize the chimera state, which is defined as

(13) $$\begin{array}{l} \omega_{k}=\frac{2\pi M_{k}}{\Delta T},\;k=1,2,\cdots \;,N \end{array}$$

where Δ*T* is the considered time interval and *M*_*k*_ is the total firing number of node-*k* in Δ*T*. Figure 2C represents the results. ω_*k*_ is a constant for coherent part but lie on a continuous curve for incoherent part. Moreover, we can also use the local order parameter to represent chimera state, defined as

(14) $$\begin{array}{l} Z_{k}=|\frac{1}{2\delta }\underset{|j-k|\leq \delta }{\sum }e^{i\theta_{j}}|,\;k=1,2,\cdots \;,N \end{array}$$

where θ_*j*_ is defined by Equation (3). The *k*-th unit is in the coherent part of the chimera state when *Z*_*k*_ = 1 and incoherent parts otherwise. Figure 2D shows the evolution of *Z*_*k*_ by choosing the window size δ = 25, where the yellow parts denote the coherent regions.

**Figure 3:**
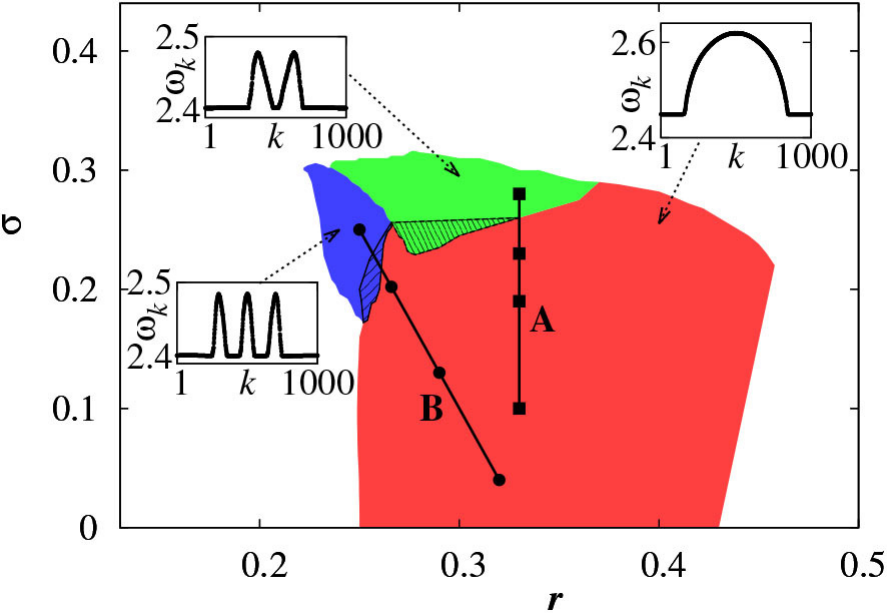
Phase diagram of chimera states on the *r* − σ plane where the red, green, and blue regions represent the chimera states with one, two and three incoherent domains, respectively. Other parameters as in Figure 2. Insets show typical profiles of the mean phase velocities. Reprinted with permission from Omelchenko et al. (2013).

For a larger coupling σ, we may observe a multichimera state where the incoherent part is divided into several independent domains, in contrast to the single incoherent domain in chimera state. Figure 3 shows the phase diagram of chimera states on the *r* − σ plane, where the red region represents the chimera state with one incoherent domain, while the green and blue regions represent the multichimera states with two or three incoherent domains, respectively.

**Figure 4:**
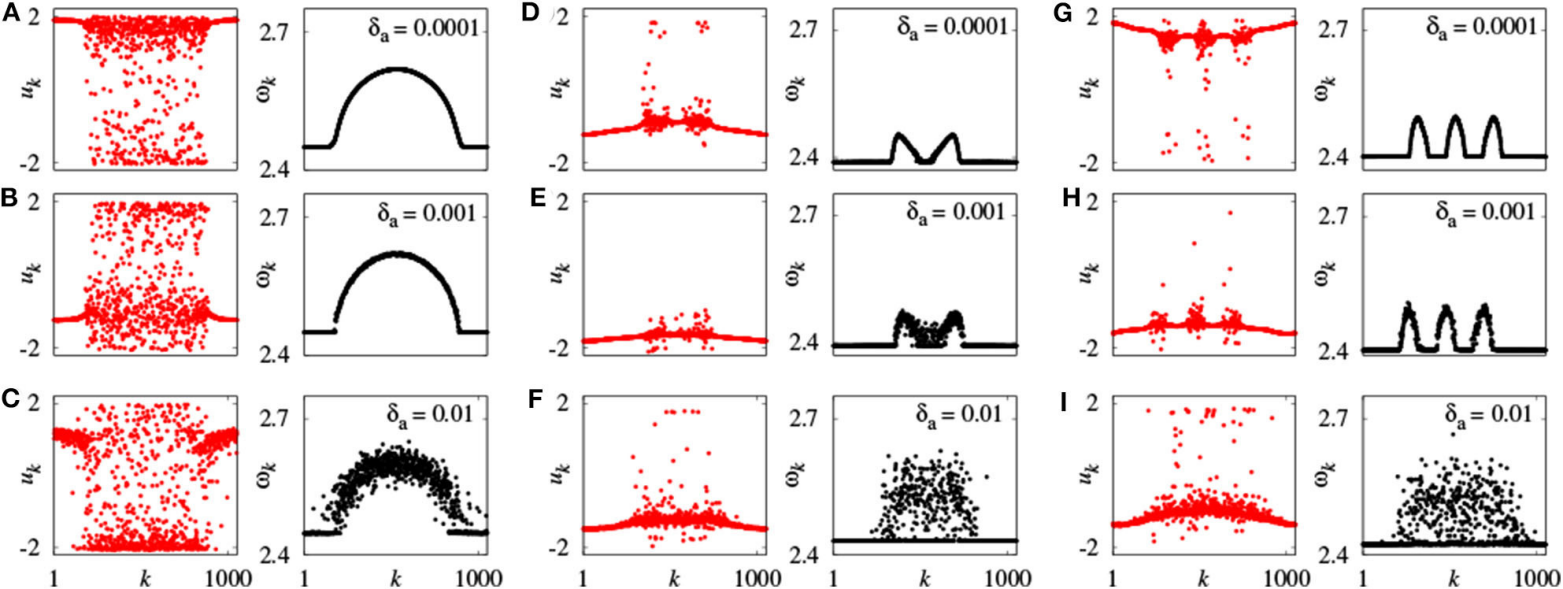
Snapshots of the variables *u*_*k*_ and mean phase velocities ω_*k*_ for inhomogeneous oscillators. **(A–C)** *r* = 0.35, σ = 0.1; **(D–F)** *r* = 0.33, σ = 0.28; **(G–I)** *r* = 0.25, σ = 0.25; values of δ*a* shown for each panel above the mean phase velocities plots. Other parameters as in Figure 2. Reprinted with permission from Omelchenko et al. (2015).

Further, Omelchenko et al. extended chimera state to the case of nonidentical FHN units (Omelchenko et al., 2015). They randomly choose the parameters *a*_*k*_ from a normal (Gaussian) distribution with mean value *a*_*mean*_ and variance δ*a*, so that the neurons have different frequencies. For fixed *a*_*mean*_ = 0.5, chimera state changes with δ*a*. Figure 4 represents a few typical snapshots and corresponding ω_*k*_ for chimera states with one, two, and three incoherent regions, as δ*a* increases. It is clear that these chimera states have some robustness to the nonidentity of oscillators (Omelchenko et al., 2015).

Semenova et al. considered the case with noise (Semenova et al., 2016), as it is unavoidable in real systems. They focus on the question whether noise is beneficial for chimera states. In fact, this consideration is related to the phenomenon of coherence resonance (Hu et al., 1993; Pikovsky and Kurths, 1997; Liu and Lai, 2001), where an optimal noise intensity can result in the counterintuitive increase of temporal coherence. Semenova et al. found that noise is essential for chimera behavior and call it *coherence-resonance chimera* (Semenova et al., 2016). That is, an optimal noise can even induce a spatial chimera state, rather than purely temporal coherence. Their model is a slight modification of Equation (11) and can be written as

(15) $$\begin{array}{l} \epsilon \frac{du_{i}}{dt}=u_{i}-\frac{u_{i}^{3}}{3}-v_{i}+\frac{\sigma }{2R}\overset{i+R}{\underset{j=i-R}{\sum }}[b_{uu}\left(u_{j}-u_{i}\right)\\ \;+b_{uv}\left(v_{j}-v_{i}\right)],\\ \frac{dv_{i}}{dt}=u_{i}+a_{i}+\frac{\sigma }{2R}\overset{i+R}{\underset{j=i-R}{\sum }}[b_{vu}\left(u_{j}-u_{i}\right)\\ \;+b_{vv}\left(v_{j}-v_{i}\right)]+\sqrt{2D}\xi_{i}\left(t\right), \end{array}$$

where ξ_*i*_(*t*) represents the Gaussian white noise with 〈ξ_*i*_(*t*)〉 = 0 and $\langle \xi_{i}\left(t\right)\xi_{j}\left(t^{\prime }\right)\rangle =\delta_{ij}\delta \left(t-t^{\prime }\right)$, and *D* denotes the noise intensity. The rotational coupling matrix and phase parameter are fixed as the same as in Equation (11) except *a*_*i*_ ≡ *a* = 1.001, indicating that all neurons are excitable but close to the threshold.

**Figure 5:**
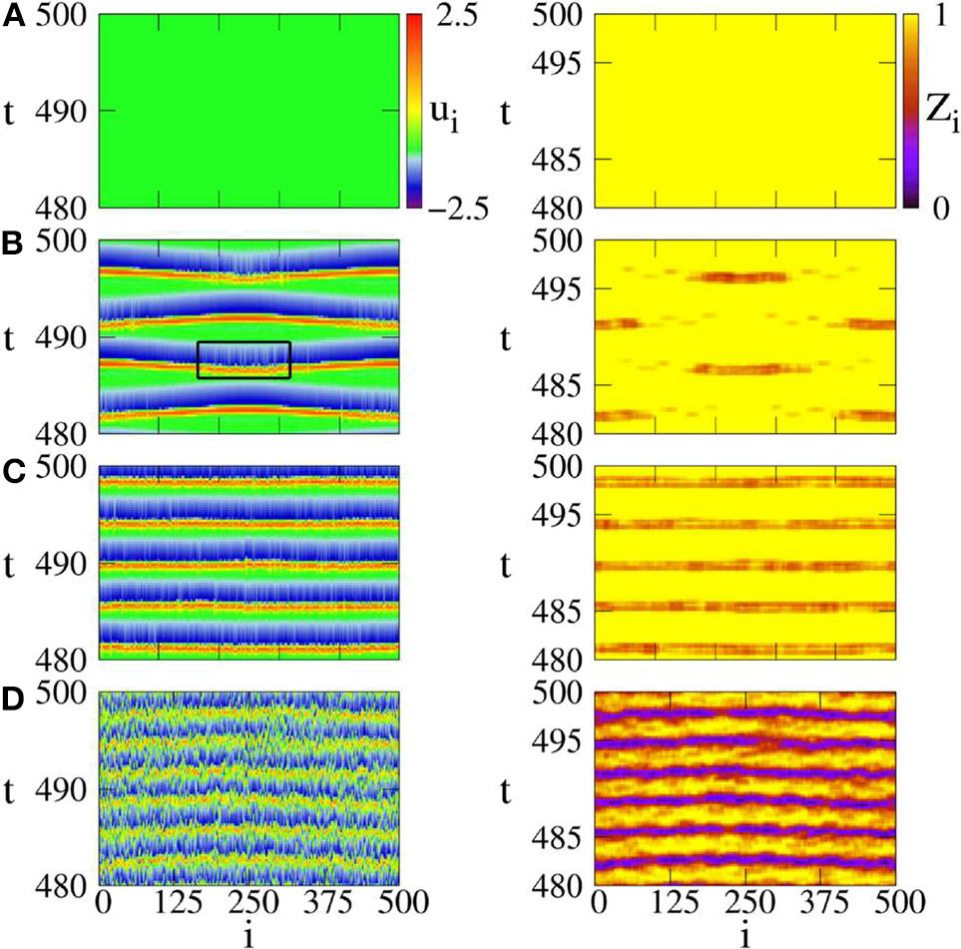
Space-time plots of activator *u*_*i*_ (left column) and local order parameter *Z*_*i*_ (right column) for different noise intensities. **(A)** *D* = 0: steady state, **(B)** *D* = 0.0002: coherence resonance chimera, **(C)** *D* = 0.0004: incoherent in space but periodic in time, **(D)** *D* = 0.1: incoherent in space and time. Parameters: ϵ = 0.05, *a* = 1.001, σ = 0.4, *r* = 0.12. Reprinted with permission from Semenova et al. (2016).

The presence of noise may induce a spike for a single FHN neuron in the excitable regime of |*a*| > 1. When the noise intensity is too small, it is not enough to induce a spike in the system of Equation (15). While a strong enough noise may induce too many spikes and results in irregular behaviors. Thus, there may exist an optimum intermediate noise to induce a chimera state. To confirm this analysis, Figure 5 shows four distinct regimes by *u*_*i*_ and *Z*_*i*_ from Equation (14). Figure 5A shows the case of *D* = 0, with no spikes. Figure 5B shows the case of intermediate noise with *D* = 0.0002. The values of *Z*_*i*_ show the characteristic feature of chimera state, i.e., the coexistence of coherent and incoherent spiking. Thus, Semenova et al. called it as *coherence-resonance chimera* (Semenova et al., 2016). Figure 5C shows the case of strong noise with *D* = 0.0004, where the coherence-resonance chimera is destroyed. Figure 5D shows the case of much stronger noise with *D* = 0.1, where the spiking is incoherent in both time and space.

**Figure 6:**
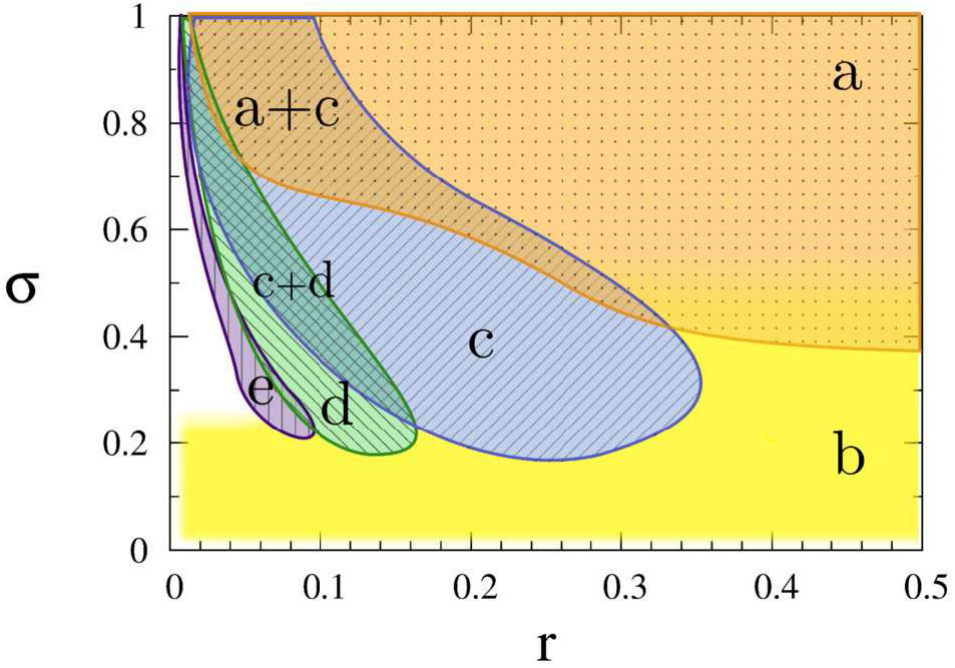
Dynamic regimes in the (*r*, σ) parameter plane: (a) steady state (orange dotted), (b) incoherent in space and periodic in time (yellow plain), (c) coherence-resonance (CR) chimera with one incoherent domain (blue crosshatched), (d) CR chimera with two incoherent domains (green crosshatched), (e) CR chimera with three incoherent domains (purple vertically hatched). Other parameters: ϵ = 0.05, *a* = 1.001, *D* = 0.0002, *N* = 500. Reprinted with permission from Semenova et al. (2016).

To see how the coupling parameters *r* and σ influence the coherence resonance chimera, Figure 6 shows the phase diagram by fixing the parameters ϵ, *a, D, N*. Region (a) represents a homogeneous steady state, region (b) shows the state of spiking patterns with temporal periodicity and spatial incoherence, regions (c–e) represent the coherence-resonance chimeras with one, two, and three incoherent domains, respectively. Therefore, except the noise intensity *D*, the coherence-resonance chimeras are also influenced by the two parameters *r* and σ.

Further, the values of *Z*_*i*_ in Figure 5B show a periodic switching between the coherent and incoherent regimes of chimera state. This feature may be helpful for the understanding of unihemispheric sleep, where the coherent and incoherent behaviors are known to switch between the two hemispheres of brain (Mukhametov et al., 1977). In fact, this kind of alternating chimera behavior has been previously addressed in a phase model by a sinusoidal signal (Ma et al., 2010), which can be also considered as an external perturbation.

Tian et al. considered the case of time-delay and electromagnetic induction in FHN neurons and found that either the time delay or electromagnetic induction can induce chimera states (Tian et al., 2018). By considering both the effect of time-delay and electromagnetic induction, Equation (11) becomes

(16) $$\begin{array}{l} \epsilon {\dot{u}}_{i}=u_{i}-\frac{u_{i}^{3}}{3}-v_{i}+\frac{\sigma }{2R}\overset{j=i+R}{\underset{j=i-R}{\sum }}\left[u_{j}\left(t-\tau \right)-u_{i}\right]\\ \;+k\rho \left(\varphi_{i}\right)u_{i},\\ {\dot{v}}_{i}=u_{i}+a,\\ {\dot{\varphi }}_{i}=k_{1}u_{i}-k_{2}\varphi_{i}, \end{array}$$

where φ represents the magnetic flux and τ denotes the time-delay. The term *kρ*(φ_*i*_)*u*_*i*_ is the induction current (Tian et al., 2018). The nonlinear function ρ(φ) is taken as ρ(φ) = α + 3βφ^2^ (Ma et al., 2017), where α and β are two parameters.

**Figure 7:**
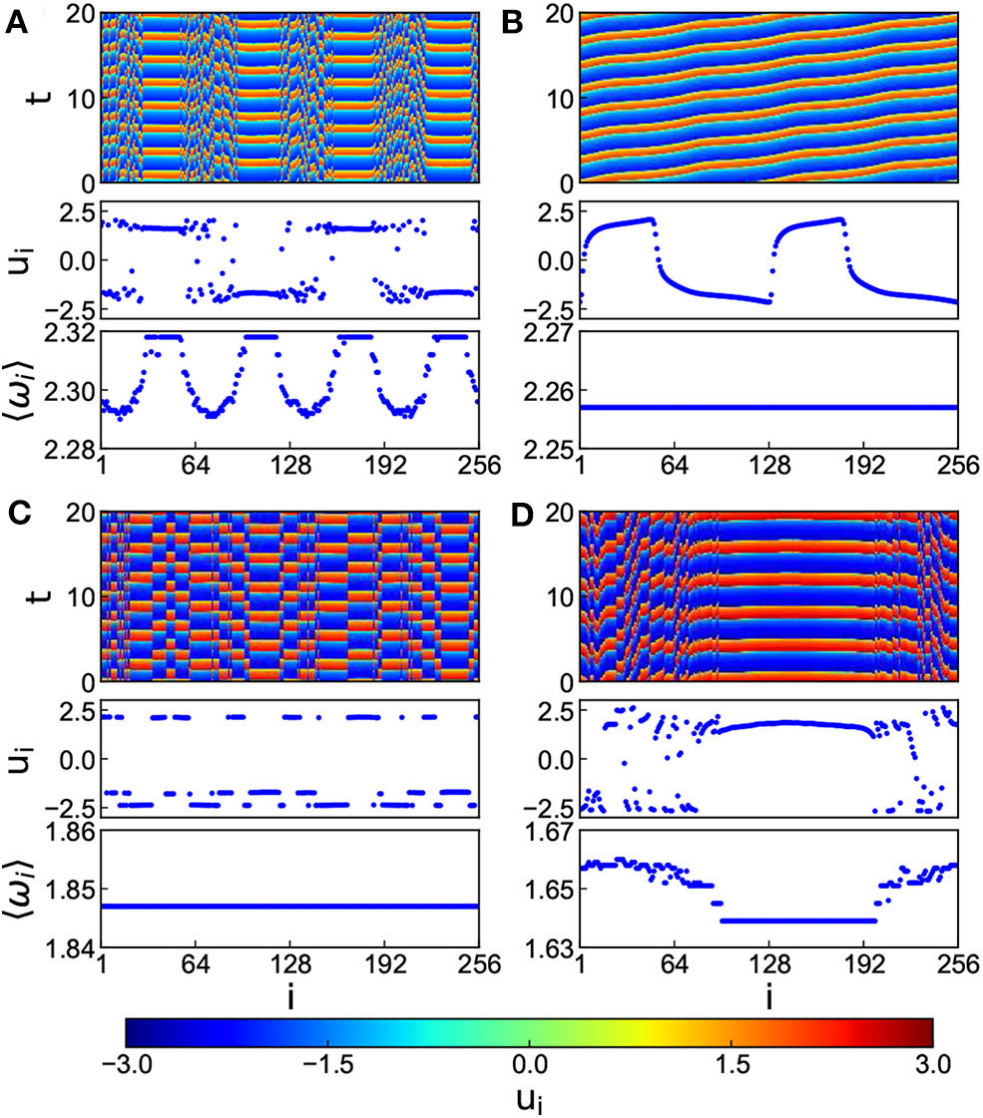
Chimera states for the situation of both time-delay and electromagnetic induction with positive *k* and *r* = 0.35, τ = 1.0, and *c* = 0.02. **(A–D)** Represent the cases of *k* = 0.8, 1.2, 5.2, and 7.4, respectively. Reprinted with permission from Tian et al. (2018).

In numerical simulations, the parameters are fixed as *N* = 256, *k*_1_ = 0.1, *k*_2_ = 1.0, α = 0.1, β = 0.1, τ = 1.0, σ = 0.02 and *r* = 0.35. Figure 7 shows the results where Figures 7A–D denote the cases of *k* = 0.8, 1.2, 5.2, and 7.4, respectively. It is clear from Figures 7A–D that with the increase of *k*, the multi-chimera state gradually becomes a chimera state.

Now, we turn to the studies of chimera state in HR neurons. Different from the FHN model of spiking neurons, HR model may represent the bursting behavior of neurons. We here concern about how this bursting behavior influences the chimera state.

Bera et al. studied a network of bursting HR neurons with global coupling as follows (Bera et al., 2016)

(17) $$\begin{array}{l} {ẋ}_{i}=ax_{i}^{2}-x_{i}^{3}-y_{i}-z_{i}+\frac{k}{N-1}\left(v_{s}-x_{i}\right)\overset{N}{\underset{j=1}{\sum }}c_{ij}\Gamma \left(x_{j}\right),\\ {ẏ}_{i}=\left(a+\alpha \right)x_{i}^{2}-y_{i},\\ {ż}_{i}=c\left(bx_{i}-z_{i}+e\right),\;i=1,2,\ldots ,N \end{array}$$

where *k* is the coupling strength, *x* is the membrane potential, and *y* and *z* are the transport of ions across the membrane through the fast and slow channels, respectively. The HR neuron is excitatory for *x*_*i*_(*t*) < *v*_*s*_, where *v*_*s*_ = 2 is the reversal potential. (*c*_*ij*_) is the adjacent matrix with *c*_*ij*_ = 1 if *i* ≠ *j* and *c*_*ii*_ = 0. The coupling function Γ(*x*) is assumed to be the sigmoidal nonlinear function

(18) $$\begin{array}{l} \Gamma \left(x\right)=\frac{1}{1+e^{-\lambda^{\prime }\left(x-\Theta_{s}\right)}}, \end{array}$$

where λ′ determines the slope of the function and Θ_*s*_ is the synaptic threshold. These two parameters are taken as Θ_*s*_ = −0.25 and λ′ = 10.

**Figure 8:**
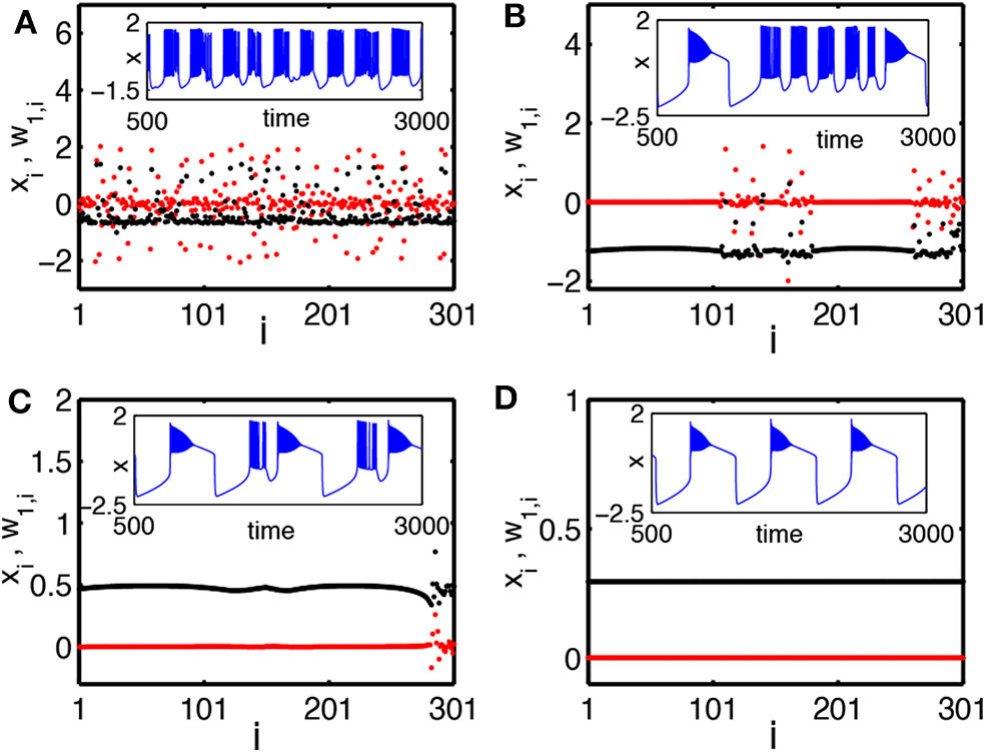
Snapshots of a system of globally coupled HR neurons for different values of the synaptic coupling strength *k* in terms of the variables *x*_*i*_ (black color) and the transformed variables *w*_1,*i*_ = *x*_*i*_ − *x*_*i*+1_ (red color): **(A)** incoherent state, *k* = 1.0; **(B)** chimera state (with two desynchronized groups), *k* = 1.2; **(C)** chimera state (with single desynchronized group), *k* = 1.28; and **(D)** coherent state, *k* = 1.3. The inset figures are the corresponding time series (blue color). The number of oscillators is *N* = 301. Reprinted with permission from Bera et al. (2016).

To characterize the chimera state, a new transformed variable *w*_1,*i*_ = *x*_*i*_ − *x*_*i*+1_ is introduced (Bera et al., 2016). Figure 8 shows the results for snapshots of the state variables *x*_*i*_ and *w*_1,*i*_ by black and red color dotted points, respectively. Figure 8A shows the case of a weak coupling *k* = 1.0. It is a disordered state. Figure 8B shows the case of a middle coupling *k* = 1.2. It is a multichimera state with two domains of disordered oscillators. The inset shows a typical time series of *x*_*i*_ (blue color line). Its behavior changes between the square-wave and plateau bursting. Figure 8C shows the case of a strong coupling *k* = 1.28. Its chimera state has only one incoherent domain, in contrast to the two incoherent domains in Figure 8B. Figure 8D shows the case of a stronger coupling *k* = 1.3, where all the neurons become coherent.

Hizanidis et al. considered a modular network of HR neurons (Hizanidis et al., 2016), which is from the *C. elegans* network and consists of six communities. They let the neurons be connected by two types of synapses: electrical and chemical. The former is for the connections within each community and the latter for the connections across the communities. Their model can be described as follows

(19) $$\begin{array}{l} {ṗ}_{i}=q_{i}-ap_{i}^{3}+bp_{i}^{2}-n_{i}+I_{\text{ext}}\\ \;+g_{el}\overset{N}{\underset{j=1}{\sum }}L_{ij}H\left(p_{j}\right)-g_{ch}\left(p_{i}-V_{\text{syn}}\right)\overset{N}{\underset{j=1}{\sum }}T_{ij}\Gamma \left(p_{j}\right),\\ {\dot{q}}_{i}=c-dp_{i}^{2}-q_{i},\\ {ṅ}_{i}=r\left[s\left(p_{i}-p_{0}\right)-n_{i}\right],\;i=1,2,\ldots ,N \end{array}$$

where *p*_*i*_ represents the membrane potential, *q*_*i*_ and *n*_*i*_ are associated with the fast and slow currents, respectively. The parameters are chosen as *r* = 0.005, *a* = 1, *V*_syn_ = 2, *b* = 3, *d* = 5, *c* = 1, *s* = 4, *p*_0_ = −1.6 and *I*_ext_ = 3.25 so that each neuron has the spiking-bursting behavior (Hizanidis et al., 2016). **L** is the Laplacian matrix with *L*_*ij*_ = *E*_*ij*_ − δ_*ij*_*k*_*i*_, where δ_*ij*_ = 1 if *i* = *j*, and δ_*ij*_ = 0 otherwise. **E** is the adjacency matrix with *E*_*ij*_ = 1 if there is an electrical synapse connecting the neurons *i* and *j*, and *E*_*ij*_ = 0 otherwise. *g*_*el*_ is the strength of the electrical coupling and its functionality is governed by the linear function *H*(*p*) = *p*. The adjacency matrix **T** is *T*_*ij*_ = 1 if there is a chemical synapse between neurons *i* and *j*, and *T*_*ij*_ = 0 otherwise. *g*_*ch*_ is the strength of the chemical coupling and its functionality is defined by the sigmoidal function Γ(*p*) from Equation (18).

**Figure 9:**
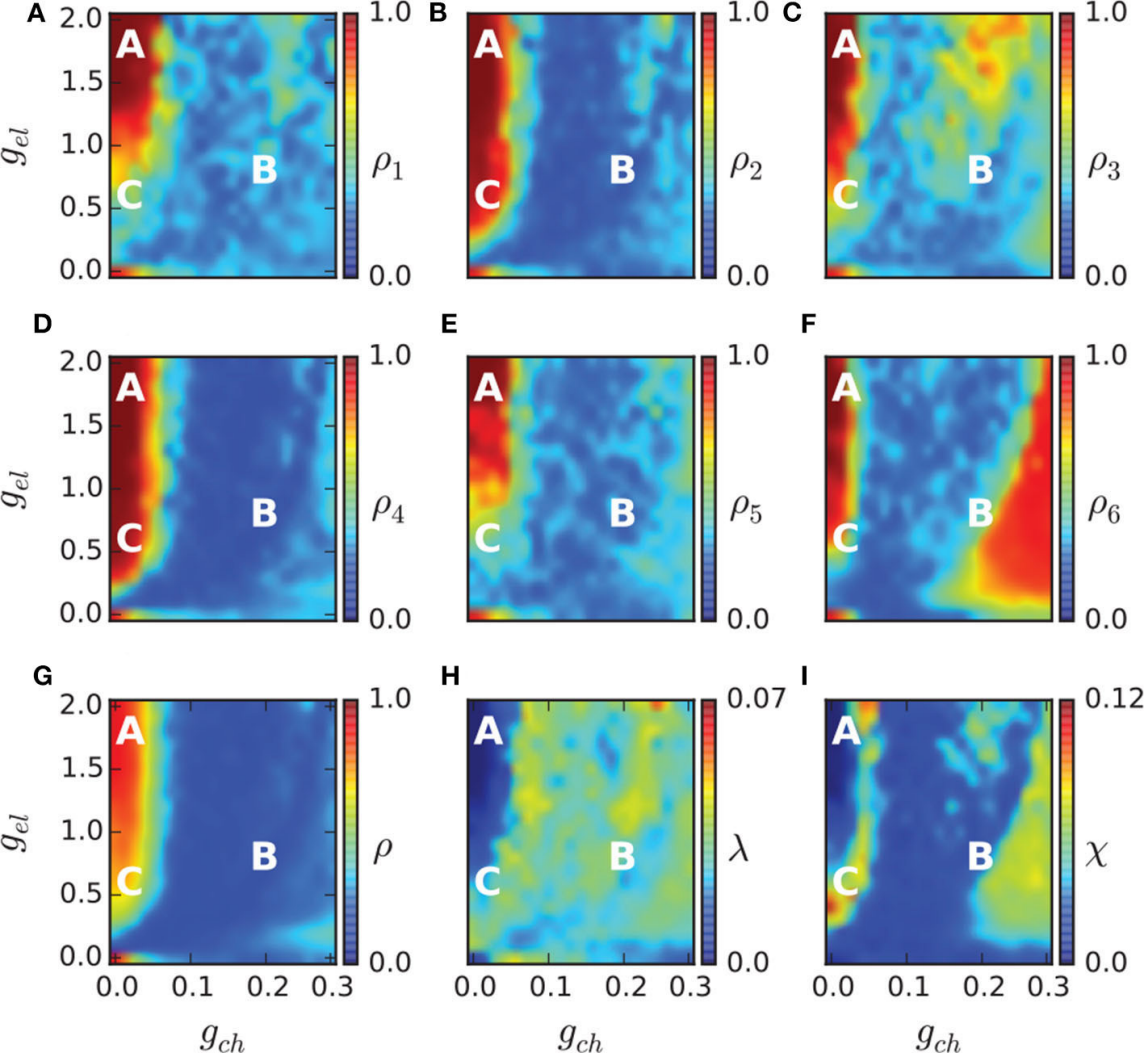
Phase diagram in the (*g*_*ch*_, *g*_*el*_) parameter spaces. The order parameter of each community ρ_1, …, 6_ is shown in **(A–F)**, and of the entire network in **(G)**. The metastability index λ is shown in **(H)** and the chimera-like index χ in **(I)**. The marked points A (*g*_*ch*_ = 0.015, *g*_*el*_ = 1.7), B (*g*_*ch*_ = 0.18, *g*_*el*_ = 0.7), and C (*g*_*ch*_ = 0.015, *g*_*el*_ = 0.5) denote three different dynamical regimes and their dynamical behaviors are illustrated in Figure 10. Reprinted with permission from Hizanidis et al. (2016).

To study the chimera state of Equation (19), Hizanidis et al. (2016) used ρ to represent the order parameter of Equation (1) where θ is calculated through Equation (4) by *p* and *q*. Figures 9A–G show the phase diagrams of ρ on the (*g*_*ch*_, *g*_*el*_) parameter space for each of the six communities and for the entire network, respectively. It is clear that ρ is not homogeneously distributed in the (*g*_*ch*_, *g*_*el*_) plane but with higher ρ in some region and lower ρ in other regions. For example, the red regions have ρ ≈ 1 and the yellow regions have 0 < ρ < 1.

**Figure 10:**
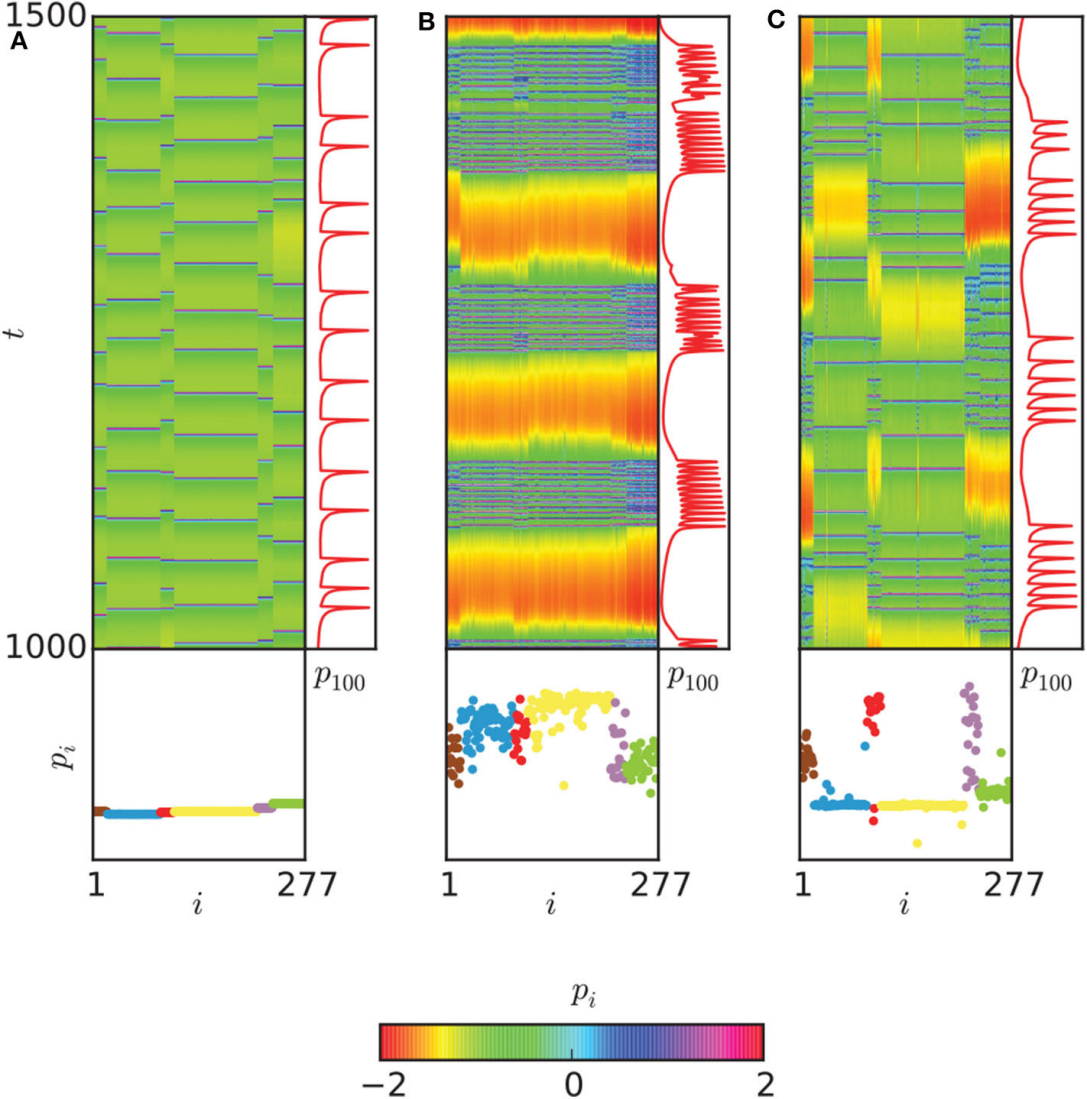
Dynamical behaviors of the three points A, B, and C in Figure 9. **(A)** The spatiotemporal evolution of *p*_*i*_ (upper left), with a time series of the neuron with index 100 of community 3 (upper right) and a snapshot of the system state (bottom) are shown for the point A of Figure 9. **(B)** The same plot for point B. **(C)** The same plot for point C. A chimera-like state is illustrated here. Neurons are ordered according to their community. Reprinted with permission from Hizanidis et al. (2016).

The regions with 0 < ρ < 1 in Figures 9A–G may represent the chimera states. To confirm it, the two measures of metastability index λ of Equation (6) and chimera-like index χ of Equation (8) are used. Figures 9H,I show the results on the (*g*_*ch*_, *g*_*el*_) parameter space. From Figure 9H we see that λ reaches higher values in some regions, implying that the system often changes between coherent and incoherent states. From Figure 9I we see that χ reaches its highest values in the two synchronization “islands” of communities 3 and 6. For more detailed information on chimera state, Hizanidis et al. chose 3 interest points on the (*g*_*ch*_, *g*_*el*_) phase diagram, marked by letters **A**, **B**, and **C** (Hizanidis et al., 2016), where **A** has both low-valued λ and χ, **B** has λ ≫ χ, and **C** has χ ≫ λ, i.e., “chimera-like” state. Figure 10 shows the dynamical behaviors of *p* for the three points. The snapshots of the system state in the bottom confirm the corresponding behaviors, where only the point **C** shows the feature of chimera state, i.e., the coexistence of coherent and incoherent domains.

Moreover, Majhi et al. studied the chimera state in uncoupled HR neurons induced by a multilayer network (Majhi et al., 2016), where the neurons in the upper layer is unconnected but can share information through the neurons in the lower layer. This topology is related to the remote synchronization (Bergner et al., 2012) and thus may help us to understand brain functions. Bera et al. reported a new type of non-stationary chimera pattern in coupled HR neurons (Bera et al., 2019), called *spike chimera*.

In summary, these studies show different ways to induce chimera states, including both cross-coupling and single-variable coupling. At the same time, these studies also show that chimera states are available in different neuronal models, indicating the robustness to neuronal models. The drawbacks or limitations are that all the considered network structures are artificial but not empirical brain networks. And the models are for individual neurons but not the average behaviors of an ensemble of neurons, which are the only available time series in experiments such as EEG data.

## 4. Chimera States in Empirical Brain Networks

Except the above extensive studies of chimera states in artificial neural systems, recently, some attention has been paid to the networks of human cerebral cortex measured by DTI. The former helps us to understand the mechanism of chimera state such as how chimera state is induced and what is the condition for chimera state to show up. While the latter highlights a way to cure or control brain diseases such as schizophrenia, Alzheimer's disease and brain tumors. It is well-known that in the fields of nonlinear science and complex network, brain functions can be represented by their corresponding dynamical patterns, i.e., a variety of patterns of partial synchronization. These patterns have a close relationship with chimera states and can be considered as a natural link between coherent and incoherent dynamics. Thus, the studies of chimera states on empirical brain networks is very helpful for exploring the mechanism of brain functions such as cognition and memory. We here make a brief summary for those results on the empirical brain networks, i.e., from the brain networks with smaller size to the middle and then to the larger ones.

Firstly, we introduce the study on an empirical brain network with smaller size. In this case, Bansal et al. considered an empirical brain network consisting of 76 brain regions (or nodes) and paid attention to how brain structure influences the dynamical patterns produced by stimulation (Bansal et al., 2019). They divided this network into nine cognitive systems by using personalized brain network models, named attention, visual, cingulo-opercular, subcortical, medial default mode, somatosensory and motor, frontoparietal, ventral temporal association, and auditory systems. Each of the nine cognitive systems is consist of the coactivated regions for supporting a generalized class of cognitive functions. Then, they presented a chimera-based, cognitively informed framework to study how large-scale brain structure influences brain dynamics and functions, called *cognitive chimera states*. In their study, the dynamics of each node was modeled by the Wilson-Cowan oscillators (Wilson and Cowan, 1972), a biologically motivated neural mass model, represented as follows

(20) $$\begin{array}{l} \tau \frac{dE_{i}}{dt}=-E_{i}\left(t\right)+\left(S_{E_{m}}-E_{i}\left(t\right)\right)S_{E}(c_{1}E_{i}\left(t\right)-\\ \;c_{2}I_{i}\left(t\right)+c_{5}\underset{j}{\sum }A_{ij}E_{j}\left(t-\tau_{d}^{ij}\right)+P_{i}\left(t\right)),\\ \tau \frac{dI_{i}}{dt}=-I_{i}\left(t\right)+\left(S_{I_{m}}-I_{i}\left(t\right)\right)S_{I}(c_{3}E_{i}\left(t\right)-\\ \;c_{4}I_{i}\left(t\right)+c_{6}\underset{j}{\sum }A_{ij}I_{j}\left(t-\tau_{d}^{ij}\right)), \end{array}$$

where

(21) $$\begin{array}{l} S_{E,I}\left(x\right)=\frac{1}{1+e^{-a_{E,I}\left(x-\theta_{E,I}\right)}}-\frac{1}{1+e^{a_{E,I}\theta_{E,I}}} \end{array}$$

and *A*_*ij*_ is the weighted coupling matrix. *c*_5_ and *c*_6_ represent the excitatory and inhibitory coupling strength, respectively, with *c*_6_ = *c*_5_/4. *P*_*i*_(*t*) is the external stimulation. $\tau_{d}^{ij}=d_{ij}/t_{d}$ is the time-delay, where *d*_*ij*_ is the spatial distance between nodes *i* and *j* and *t*_*d*_ = 10*m*/*s* is the signal transmission speed. Other parameters are biologically taken as *c*_1_ = 16, *c*_2_ = 12, *c*_3_ = 15, *c*_4_ = 3, τ = 8, θ_*I*_ = 3.7, θ_*E*_ = 4, *a*_*I*_ = 2, and *a*_*E*_ = 1.3.

In numerical simulations, the dynamical behaviors of the nine cognitive systems were investigated by stimulating a brain region with *P*_*i*_ = 1.15 (Bansal et al., 2019). The stimulation gradually spreads to other parts of the brain network by the links of the stimulated node and form a dynamical state. By this way, different dynamical states are observed when different brain regions are stimulated. Then, Bansal et al. calculated a cognitive system-based order parameter ρ_*si,sj*_ from Equation (1). This parameter ρ_*si,sj*_ measures the degree of synchrony among all oscillators within the two cognitive systems *s*_*i*_ and *s*_*j*_. By this way, a cognitive system-based 9 × 9 matrix can be obtained. Further, two cognitive systems *s*_*i*_ and *s*_*j*_ are considered as synchronized if ρ_*si,sj*_ exceeds a threshold value ρ_*Th*_ = 0.8. The whole brain network is a coherent state when all nine cognitive systems are synchronized, a cognitive chimera state when some cognitive systems are synchronized while the other systems are desynchronized, and a metastable state when no stable synchrony between cognitive systems is formed (Bansal et al., 2019).

**Figure 11:**
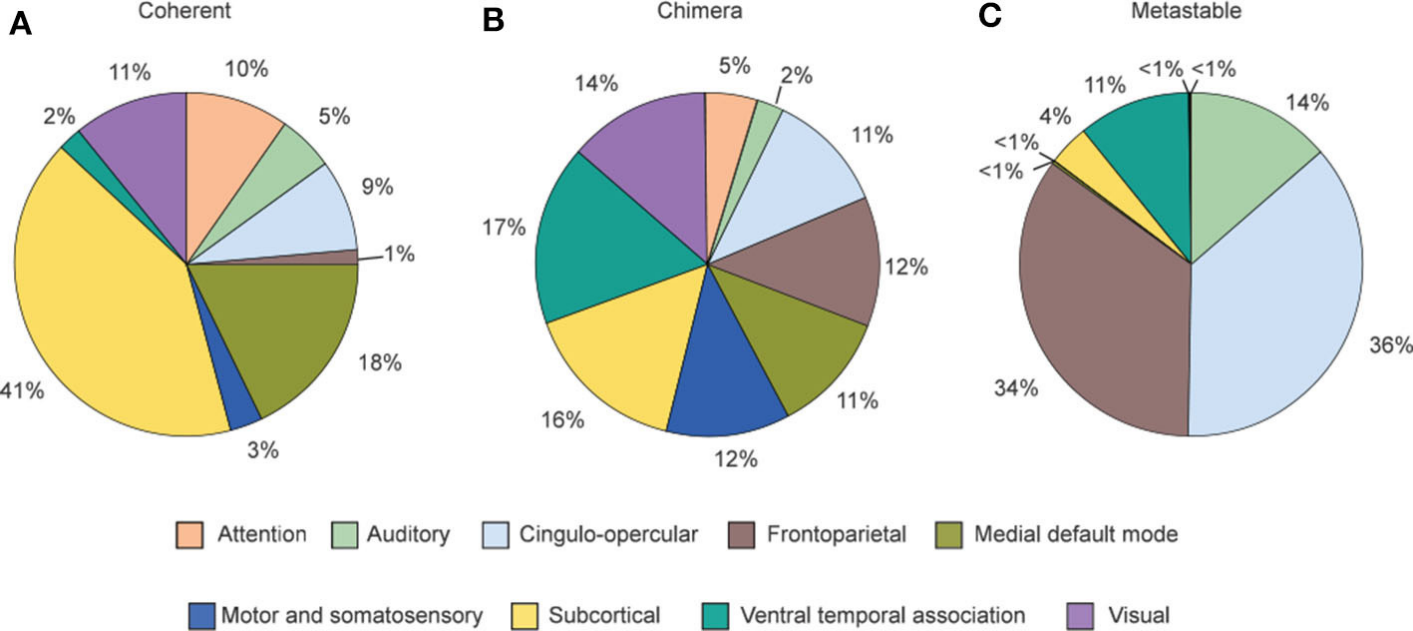
Distribution of the contribution fractions from the nine cognitive systems after nodes stimulation where **(A–C)** represent the cases of final dynamical states as coherent, chimera and metastable states, respectively. This distribution tells a relationship between the dynamical states and individual cognitive systems. Reprinted with permission from Bansal et al. (2019).

Figure 11 shows the relative contribution of the nine cognitive systems for the three states after nodes stimulation. We see that the contributions to the three states are different from one cognitive system to another, implying a close relationship between cognitive systems and brain functions. Figure 11A shows that coherent states are mainly from the nodes stimulation within subcortical and medial default mode systems. Figure 11C shows that the frontoparietal and cingulo-opercular systems are the main contributions for metastable states, while the ventral temporal association and auditory systems also contribute substantially to metastable states. Figure 11B shows that all nine systems produce chimera states, implying that chimera states have higher possibility to be observed than either coherent or metastable states (Bansal et al., 2019). Bansal et al. also pointed that the regions of coherent states are distributed more closely to the midline of the brain, and the regions of metastable states are distributed farther from the midline, i.e., along the edges of the hemispheres, while the regions of chimera states are relatively uniformly distributed within the brain space. The metastable states enable segregated neural processing, while coherent states enable integrated neural processing. As the brain system must integrate information across spatially distributed, segregated regions to implement cognitive tasks, a balance between integration and segregation is required for adaptive cognition. This balance is automatically satisfied in chimera states and thus enables segregation and integration in brain dynamics, which guarantees the diverse processing requirements.

Secondly, Chouzouris et al. considered a slightly larger empirical brain network consisting of 90 nodes (Chouzouris et al., 2018), motivated by studies of epileptic seizures. They let the neurons be the FitzHugh-Nagumo oscillators. As this empirical brain network has a topology of complex network, Equation (11) has to be modified into

(22) $$\begin{array}{l} \epsilon \frac{du_{k}}{dt}=u_{k}-\frac{u_{k}^{3}}{3}-v_{k}+\sigma \overset{N}{\underset{j=1}{\sum }}G_{kj}[b_{uu}\left(u_{j}-u_{k}\right)\\ \;+b_{uv}\left(v_{j}-v_{k}\right)],\\ \frac{dv_{k}}{dt}=u_{k}+a+\sigma \overset{N}{\underset{j=1}{\sum }}G_{kj}[b_{vu}\left(u_{j}-u_{k}\right)\\ \;+b_{vv}\left(v_{j}-v_{k}\right)], \end{array}$$

where ϵ = 0.05 and *G* is the adjacency matrix. The rotational coupling matrix *B* = (*b*_*uu*_) is taken as the same as in Equation (12).

**Figure 12:**
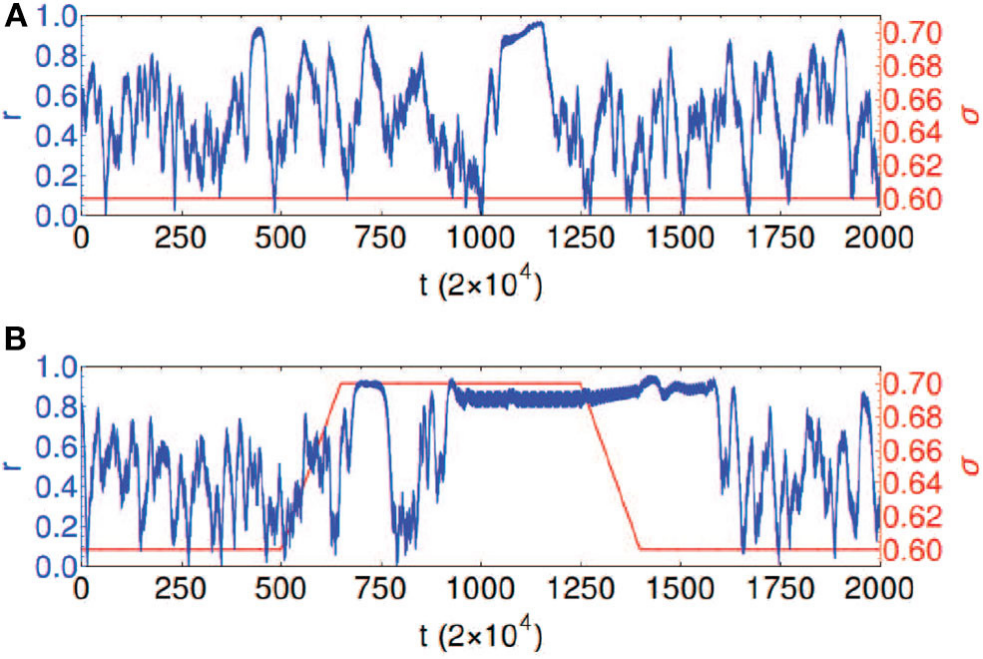
Temporal evolution of the global order parameter *r* shown in blue for the network with empirical structural connectivity with *a* = 0.5 and *N* = 90. The coupling strength σ is shown in red. **(A)** Chimera state: constant coupling strength σ = 0.6. **(B)** Controlled dynamics: coupling strength σ = 0.6 is increased to the value σ = 0.7 and kept fixed for the time interval 650 < *t*/2 × 10^4^ < 1, 350, followed by a decrease back to σ = 0.6; this causes the transitions between the chimera state and frequency synchronized states. Reprinted with permission from Chouzouris et al. (2018).

Chouzouris et al. found that when *a* is in the range *a* ∈ (0, 0.8), the network exhibits chimera states for small coupling strength σ (Chouzouris et al., 2018). When the parameters are taken as *a* = 0.5, σ = 0.2, and *N* = 90, the network shows a *stationary moving chimera*. That is, the order parameter *r* from Equation (1) has a strong fluctuation or changes in time. Figure 12A shows such an example, where the parameters are *a* = 0.5 and σ = 0.6. The average of order parameter is 〈*r*〉 ≈ 0.5. Except the strong fluctuation, another feature is that the highest value of *r* appears right before its drop. Both effects were discovered in the synchronization of epileptic seizures (Jiruska et al., 2013). Chouzouris et al. further pointed out that the high coherence events can be controlled (Chouzouris et al., 2018). Larger σ increases the probability for chimera states to occur. Figure 12B shows the result that the variation of coupling results in a switching between the chimera state and synchronization, which controls the epileptic seizures.

This empirical brain network of 90 nodes was further studied by Ramlow et al. (2019), where a dynamical asymmetry between the hemispheres was addressed by considering natural structural asymmetry.

Finally, we discuss the situation of empirical brain network with larger size. This network is much larger than the above two and is constructed by the data of Hagmann et al. (2008) where the cerebral cortex was divided into 989 nodes and 17, 865 connections. For this network, Huo et al. first considered the case of adaptive coupling (Huo et al., 2019), based on the fact that in empirical brain network, both the coupling strength and neural activities influence each other and thus change with time. In Huo's model, the coupling matrix is adaptively evolved with the dynamics of neurons. They found that the adaptive coupling finally reaches a self-organized state and induces chimera states. This kind of self-organization may support the high flexibility of brain functions. In details, they let the nodes be the FitzHugh-Nagumo oscillators and the dynamics be represented as follows

(23) $$\begin{array}{l} \epsilon {\dot{u}}_{i}=u_{i}-\frac{u_{i}^{3}}{3}-v_{i}\left(t\right)\\ \;+\frac{1}{N}\overset{N}{\underset{j=1}{\sum }}\lambda_{ij}\left[b_{uu}\left(u_{j}-u_{i}\right)+b_{uv}\left(v_{j}-v_{i}\right)\right],\\ {\dot{v}}_{i}=u_{i}+a+\frac{1}{N}\overset{N}{\underset{j=1}{\sum }}\lambda_{ij}\left[b_{vu}\left(u_{j}-u_{i}\right)+b_{vv}\left(v_{j}-v_{i}\right)\right], \end{array}$$

where ϵ = 0.05, *a* = 0.5, and the rotational coupling matrix *B* is defined as in Equation (12). The adaptive coupling λ_*ij*_ is set as

(24) $$\begin{array}{l} {\dot{\lambda }}_{ij}=-\gamma \left[\sin\left(u_{j}-u_{i}+\beta \right)+\lambda_{ij}\right], \end{array}$$

where γ is a small constant. Equation (24) does not influence the topology of network but only change the value of λ_*ij*_.

**Figure 13:**
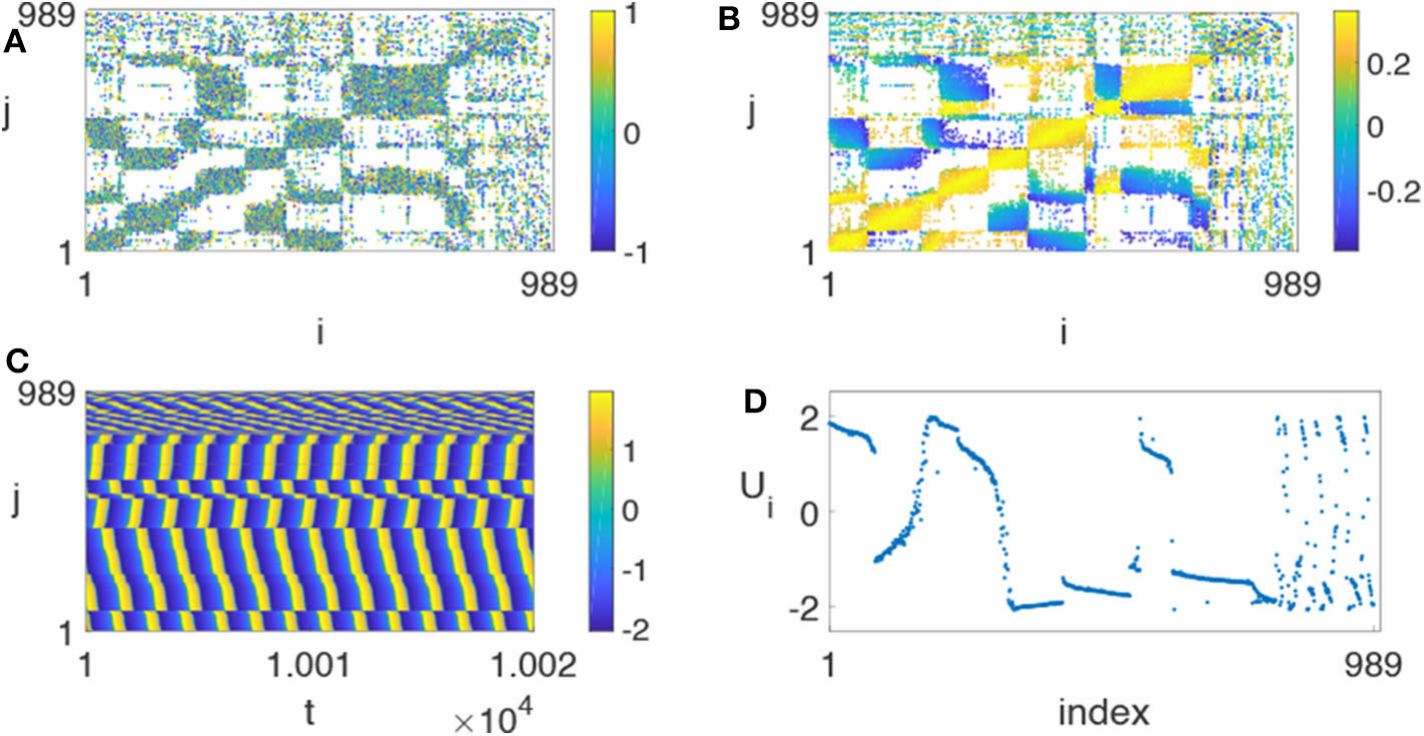
Chimera state on the realistic network of cerebral cortex with *N* = 989, ϕ = −π + 4.45 and β = −0.5π + 1.2. **(A)** Represents the initial matrix λ_*ij*_ chosen randomly from [−1, 1], **(B)** the stabilized matrix λ_*ij*_, **(C)** the evolutionary pattern of *u*_*i*_(*t*), and **(D)** the snapshot of the fast variable *u*_*i*_(*t*) at a specific time *t*. Reprinted with permission from Huo et al. (2019).

Huo et al. chose γ = 0.01 and found that it is possible for chimera states to appear in this realistic network (Huo et al., 2019). Figure 13 represents the resulted chimera state for β = −0.5π + 1.2 and ϕ = −π + 4.45, where (Figure 13A) is the initial matrix of λ_*ij*_, (Figure 13B) the stabilized matrix of λ_*ij*_, (Figure 13C) the evolutionary pattern of *u*_*i*_(*t*), and (Figure 13D) a snapshot of the fast variable *u*_*i*_(*t*). Comparing Figure 13A with (Figure 13B), we see that the stabilized matrix λ_*ij*_ in (Figure 13B) is significantly different from the initial matrix λ_*ij*_ in (Figure 13A). This may help us to understand how the brain network is self-organized into chimera states.

**Figure 14:**
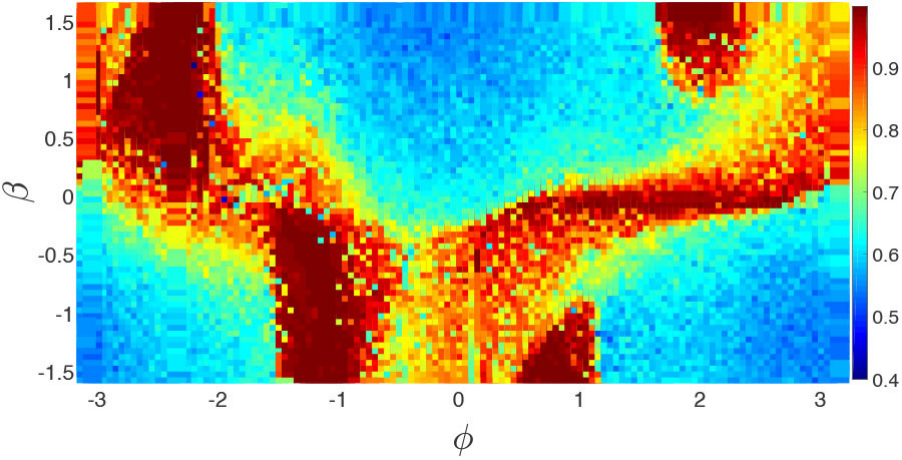
Phase diagram of *g*_0_ of chimera state in the parameter plane of ϕ and β for the realistic network of cerebral cortex. Reprinted with permission from Huo et al. (2019).

To reflect the dependence of chimera states on the parameters ϕ and β, Huo et al. calculated the measure *g*_0_ of chimera state by Equation (10). Figure 14 shows its phase diagram on the parameter plane of ϕ and β. The stabilized behaviors consist of disorder, coherent, and chimera states but the fraction of chimera states is the largest one on the phase diagram.

Kang et al. further considered the empirical brain network of 989 nodes as a two-layered network where the left and right hemispheres of cerebral cortex are considered as different layers, respectively (Kang et al., 2019). In their model, the intra- and inter-coupling strengths are considered to be different. Very interesting, they found that the model can reproduce the phenomenon of unihemispheric sleep where one hemisphere is completely synchronized while the other is completely desynchronized. This finding provides an explanation for the *first-night effect* in human sleep (Tamaki et al., 2016). Their model for the first layer-*A* is represented as follows

(25) $$\begin{array}{l} \epsilon {\dot{u}}_{i}^{a}=u_{i}^{a}-\frac{{\left(u_{i}^{a}\right)}^{3}}{3}-v_{i}^{a}\\ +\frac{\lambda_{in}}{k_{in,i}^{a}}\overset{N}{\underset{j=1}{\sum }}A_{ij}\left[d_{uu}\left(u_{j}^{a}-u_{i}^{a}\right)+d_{uv}\left(v_{j}^{a}-v_{i}^{a}\right)\right]\\ +\frac{\lambda_{out}}{k_{out,i}^{a}}\overset{N}{\underset{j=1}{\sum }}{\left(AB\right)}_{ij}\left[d_{uu}\left(u_{j}^{b}-u_{i}^{a}\right)+d_{uv}\left(v_{j}^{b}-v_{i}^{a}\right)\right],\\ {\dot{v}}_{i}^{a}=u_{i}^{a}+a\\ +\frac{\lambda_{in}}{k_{in,i}^{a}}\overset{N}{\underset{j=1}{\sum }}A_{ij}\left[d_{vu}\left(u_{j}^{a}-u_{i}^{a}\right)+d_{vv}\left(v_{j}^{a}-v_{i}^{a}\right)\right]\\ +\frac{\lambda_{out}}{k_{out,i}^{a}}\overset{N}{\underset{j=1}{\sum }}{\left(AB\right)}_{ij}\left[d_{vu}\left(u_{j}^{b}-u_{i}^{a}\right)+d_{vv}\left(v_{j}^{b}-v_{i}^{a}\right)\right], \end{array}$$

where ϵ = 0.05 and *a* = 0.5. $k_{out,i}^{a}$ and $k_{in,i}^{a}$ are the inter- and intra-degrees of node *i*, respectively. (*AB*)_*ij*_ and *A*_*ij*_ denote the inter- and intra-coupling matrices, respectively. The other quantities are the same as in Equations (11) and (12).

The similar dynamics equations can be written for the network *B*.

**Figure 15:**
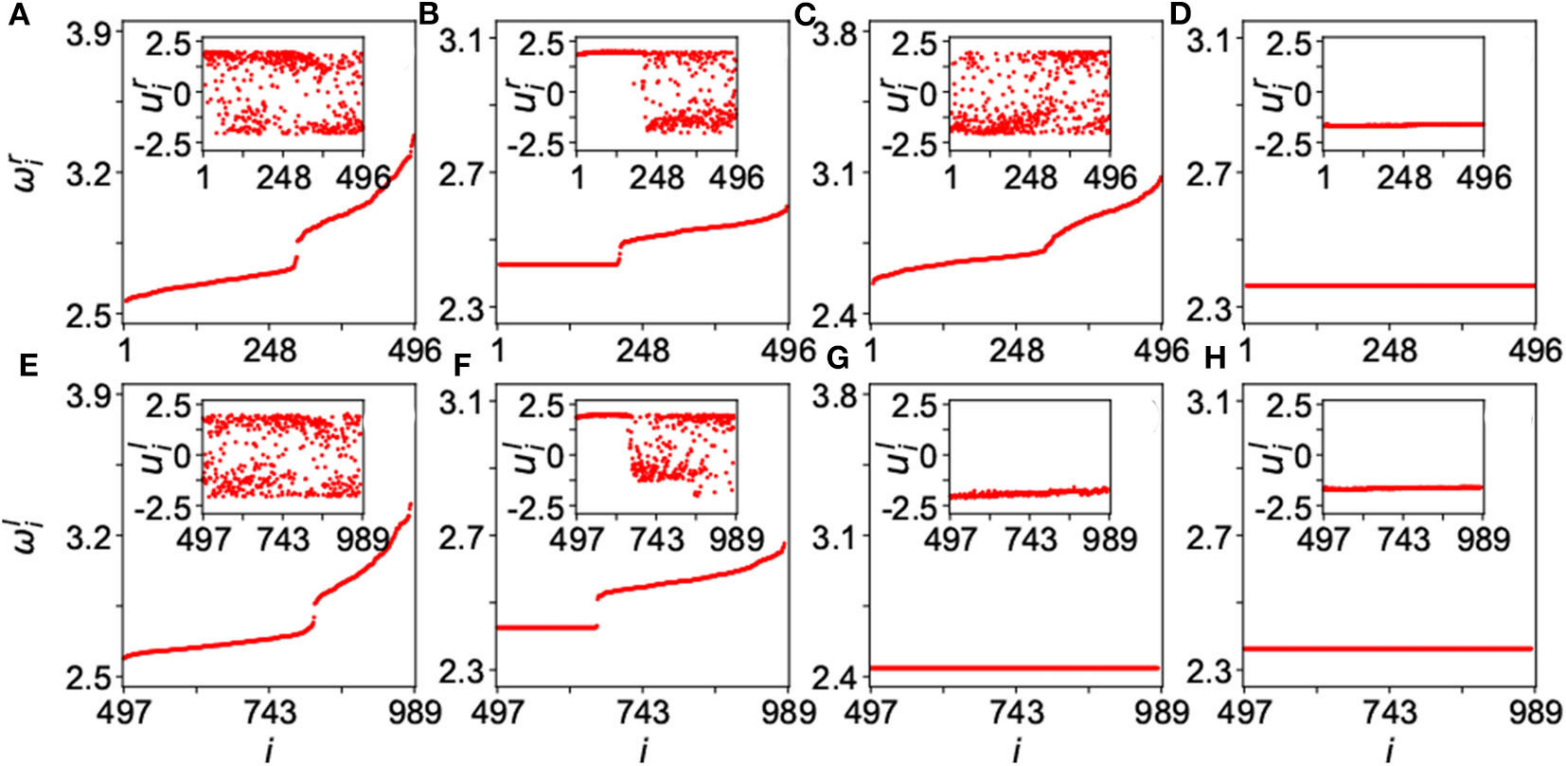
Four typical dynamical states in the two-layered network model of Equation (25) where the up panels represent the layer-A and down panels the layer-B. The inset in each panel is a snapshot of *u*_*i*_ at time *t*. **(A,E)** Represent the case of disorder with λ_*in*_ = 0.1 and λ_*out*_ = 0.3; **(B,F)** the case of chimera state with λ_*in*_ = 0.1 and λ_*out*_ = 1.8; **(C,G)** the case of unihemispheric sleep with λ_*in*_ = 0.4 and λ_*out*_ = 3.5; and **(D,H)** the case of synchronization with λ_*in*_ = 4.0 and λ_*out*_ = 3.5. Reprinted with permission from Kang et al. (2019).

Figure 15 shows the results for four typical cases where ω_*i*_ is calculated by Equation (13), the up and down panels are for the network-A and network-B, respectively, and the insets are their corresponding dynamics of *u*_*i*_. The panels of Figure 15 represent four typical cases where (Figures 15A,E) are for the case of disorder with λ_*in*_ = 0.1 and λ_*out*_ = 0.3; (Figures 15B,F) for the case of chimera state with λ_*in*_ = 0.1 and λ_*out*_ = 1.8; (Figures 15C,G) for the case of disordered network-A and synchronized network-B, with λ_*in*_ = 0.4 and λ_*out*_ = 3.5; and (Figures 15D,H) for the case of synchronization with λ_*in*_ = 4.0 and λ_*out*_ = 3.5. These four cases represent different states. The first case of Figures 15A,E and the last case of Figures 15D,H denote the two extreme states of desynchronized and synchronized states, respectively. The second case of Figures 15B,F represents a chimera state where there is a plateau of ω_*i*_ in both the up and down panels and their insets show a coexistence of synchronized and unsynchronized *u*_*i*_(*t*). The most interesting is the third case of Figures 15C,G where the network-A is disordered but the network-B is synchronized, marking the unihemispheric sleep. Kang et al. further showed that the parameter region for the state of unihemispheric sleep is much smaller than that of chimera state, implying that it is usually difficult to observe the phenomenon of unihemispheric sleep. This is consistent with the *first-night effect* (Tamaki et al., 2016), which can be observed only in the first-night sleep when a person is located in an unfamiliar place.

In sum, these studies showed the recent progress of chimera states in empirical brain networks, but did not pay much attention to the aspects of characteristic features of brain networks, such as the heterogeneous communities and hub nodes of rich-club, and deeper connection to concrete brain functions, such as cognitive and memory etc.

## 5. Discussions

Chimera state is in fact one of the three kinds of partial synchronization. The other two of them are the cluster synchronization and remote synchronization. Cluster synchronization represents the case where the oscillators of network are automatically evolved into different synchronized clusters but the oscillators in different clusters are not synchronized each other (Schaub et al., 2016; Cao et al., 2018). The relationship between the synchronized cluster and network symmetry is discussed recently (Pecora et al., 2014; Sorrentino et al., 2016), including the case where the synchronized cluster is not directly from the symmetry but due to the same total amounts of inputs received from their neighboring nodes (Siddique et al., 2018). While remote synchronization represents the synchrony among the leaf nodes of a hub but not synchronized with the hub, i.e., the synchronized nodes are not directly connected (Bergner et al., 2012). However, these three partial synchronization are not completely independent of each other but may sometimes represent the same phenomenon. For example, chimera state can appear simultaneously with cluster synchronization in some systems (Hart et al., 2016; Cho et al., 2017; Bansal et al., 2019). It is also possible for remote synchronization to be related to cluster synchronization (Bergner et al., 2012; Kang et al., 2020; Wang and Liu, 2020). For example, in a star network with remote synchronization, if the leaf nodes of the hub is considered as a cluster, their synchronization is in fact the cluster synchronization. Based on these results, it is an open but promising direction to highlight the mechanisms of brain functions such as cognition, memory, and signal spreading etc, from these three aspects of partial synchronization.

One purpose of studying chimera states in neural systems is for its potential applications. On one hand, some attention has been paid to the phenomenon of *unihemispheric sleep* (Rattenborg et al., 2000; Ma et al., 2010). On the other hand, the study of chimera state may help us to understand neuronal diseases such as epileptic seizures, Parkinson's disease, schizophrenia, Alzheimer's disease and brain tumors (Uhlhaas and Singer, 2006). For example, a therapy for Parkinson's disease is external electric stimulation at high frequencies, called deep brain stimulation (Benabid et al., 1991). These studies are still very primary. More deeper studies are expected, such as the connections of chimera states to the mechanisms of cognitive and memory and the control of various brain diseases in clinical medicine etc.

## Author Contributions

ZL and ZW conceived the research project and wrote the paper. All authors contributed to the article and approved the submitted version.

## Conflict of Interest

The authors declare that the research was conducted in the absence of any commercial or financial relationships that could be construed as a potential conflict of interest.
